# A Novel Swarm Intelligence Algorithm with a Parasitism-Relation-Based Structure for Mobile Robot Path Planning

**DOI:** 10.3390/s23041751

**Published:** 2023-02-04

**Authors:** Hui Ren, Luli Gao, Xiaochen Shen, Mengnan Li, Wei Jiang

**Affiliations:** 1 School of Information and Communication Engineering, Communication University of China, No.1 Dingfuzhuang East Street, Chaoyang District, Beijing 100024, China; 2 State Key Laboratory of Media Convergence of Communication, Communication University of China, Beijing 100024, China; 3 Key Laboratory of Acoustic Visual Technology and Intelligent Control System, Ministry of Culture and Tourism, Beijing 100024, China

**Keywords:** Para-PSO-ABC algorithm, dual-community-evolutionary structure, parasitic relationship, path planning, multi-swarm evolution

## Abstract

A multi-swarm-evolutionary structure based on the parasitic relationship in the biosphere is proposed in this paper and, according to the conception, the Para-PSO-ABC algorithm (ParaPA), combined with merits of the modified particle swarm optimization (MPSO) and artificial bee colony algorithm (ABC), is conducted with the multimodal routing strategy to enhance the safety and the cost issue for the mobile robot path planning problem. The evolution is divided into three stages, where the first is the independent evolutionary stage, with the same evolution strategies for each swarm. The second is the fusion stage, in which individuals are evolved hierarchically in the parasitism structure. Finally, in the interaction stage, a multi-swarm-elite strategy is used to filter the information through a predefined cross function among swarms. Meanwhile, the segment obstacle-avoiding strategy is proposed to accelerate the searching speed with two fitness functions. The best path is selected according to the performance on the safety and consumption issues. The introduced algorithm is examined with different obstacle allocations and simulated in the real routing environment compared with some typical algorithms. The results verify the productiveness of the parasitism-relation-based structure and the stage-based evolution strategy in path planning.

## 1. Introduction

With the development of control technology, mobile robotics has been developed in multiple fields, such as rescue, military, industry, etc. As one of the essential parts of the mobile robotics field, the path planning problem establishes an effective path for the robot to reach the target and finish the task without any collisions based on the specific environment. According to the preknowledge of the working environment, the path planning approach can be classified into two parts, which are the global path planning with static information and the local planning based on the sensor information. The former one needs to find a suitable route according to previous map information. While the local aspect should have the decision capacity upon real-time information and find where the problem is, such as the local obstacle distribution, so as to optimize a solution from the current node to a sub-target until the mission is completed. Successful planning should meet both optimization criteria in terms of time and traveling distance, etc.

**Figure 1:**
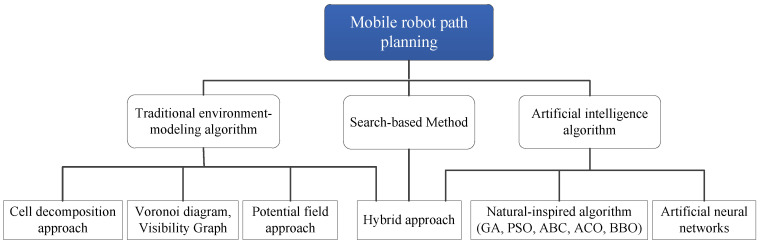
Classification of mobile robot path planning approaches.

Recently, various methods of intelligent planning have been studied to find the most productive solution. All the methods can be classified into traditional path-planning methods based on environment modeling, search-based method, and artificial intelligence algorithm (seen in Figure 1). The traditional path planning algorithms are built with the previously defined map, and most of them require environment information in advance to guide robot movement [1] or make a mobility prediction [2]. It is normally utilized to solve a global problem, such as the potential field category, which produces an artificial field based on the motion environment. The movement of the robot can be guided by descent direction, such as gravity, to avoid the repulsive fields (obstacle) from the start to the target. However, it cannot guarantee the path is the global best, even for a certain search range. Hence, the optimization algorithm is normally utilized to optimize the path generated by the artificial potential field (APF), so as to increase the effectiveness of the hybrid algorithm [1]. Another category is the search-based method, such as Dijkstra and A* algorithm, whose complexities increase with the dimensions of problems, resulting in lower effectiveness [3]. Further, a dynamic environmental problem is hard to deal with for those methods that may increase the cost of the planning. Moreover, the environment-modeling-based method, such as the Voronoi Diagram [4], Visibility Graph [5], and Cell Decomposition [6], decomposes the environment into several regions and transforms the complex workspace into a simple map search problem. This type of algorithm has a strong ability to guarantee safety, but the local optima cannot be avoided.

The artificial intelligence algorithm is probably the most commonly adopted approach for mobile robot path planning, which transforms the best path planning into a constrained optimization with one or several objective functions. The optimization algorithm is utilized to solve the nonlinear and multi-constrained models for an optimum or approximate solution, such as particle swarm optimization (PSO), ant colony optimization (ACO), artificial bee colony algorithm (ABC), genetic algorithm (GA), artificial neural network [7,8,9,10,11,12], etc. They have a great ability to handle the uncertainty condition in the complex environment and are flexible for global or local routing problems. However, the performance of this kind of method is unstable due to defects in the algorithm, such as the premature problem and the balance between exploration and exploitation, which may cause inferior work efficiency with a redundant computation. Therefore, the algorithm should be modified according to its merits. For instance, to address the slow convergence problem in the GA, Hong et al. introduced a co-evaluation strategy to provide some margin of error during evolution. Similarly, Ref. [13] proposed a parallel strategy in ACO to address the premature problem. The hybrid algorithm is another productive and flexible method that can merge the advantages of each algorithm to find the best path. Ref. [14] introduced a hybrid algorithm that combined biogeography-based optimization (BBO) and PSO based on the Voronoi diagram to deal with static path planning. Normally, a traditional path planning algorithm could generate a more stable static path. Ref. [15] utilized the merit of the potential field method, combined with a bacterial evolutionary algorithm, to reduce the disadvantages of the intelligence algorithm when it is applied in an environment with a dynamic condition. The traditional map-based method is applied with the evolution algorithm is also an effective method. Ref. [16] used fuzzy logic to enhance the searchability for a dynamic path optimization based on the A* and GA. In addition, the hybrid method can extract some special evolution mechanisms to compensate for the defect in the iteration process. Ref. [17] pointed out that the parameters in the bat algorithm (BA) can be optimized by PSO in multi-objective optimization. In this category, however, the robot always moves precisely along the predetermined path [18]. In the case of an accident, the original path would be affected and redesigned with a local search method. Hence, the falling into a local optimum and computational complexities problems of the intelligence algorithm is trickier, so as to reduce the robustness and working safety. The convergence recourse should be regrouped and allocated to balance exploration and exploitation. Most swarm intelligence algorithms are improved by the evolutionary approach, such as the whale algorithm [19] and the bat algorithm [20], which changes the form of evolution. However, such modification cannot solve the search balance problem. The other method is to construct evolutionary structures, such as ABC [21], artificial fish algorithm [22], or wolf colony algorithm [23]. Although these algorithms can effectively improve the efficiency of population utilization by allocating responsibilities to the populations, they still have inevitable problems, such as the weak local exploitation ability in the ABC. The reasons are that it cannot control the evolutionary direction and the nectar replacement mechanism, resulting in a lower convergence accuracy.

In this paper, a conception of the dual-community-evolutionary structure inspired by the parasitism relation is proposed to balance the exploration and exploitation in optimization. Then, the parasitism-relation-based algorithm, composed of the PSO and ABC algorithm (ParaPA), is applied tp the path planning problem. In the paradigm algorithm, the memory swarm in PSO is utilized to prevent the loss of optima at the superior level, while the evolution approach in ABC is tailored to maximize the convergence-resource usage at the bottom level. The swarm intelligence algorithm is a search optimization based on probability where a more uniform distribution at an earlier stage obtains a better optimal solution easier. Hence, during the evolution, the initialization phase uses a chaos-based logistic map to create a chaotic status in the first stage. Then, the personal best particles in PSO are selected in the superior population, whereas the global best is produced by the nectar selection method in the ABC process. Meanwhile, the environment is divided into several segments to decrease the dimension of the variable, which improves the adaptability of the algorithm. In addition, multi-swarm evolution with the elitist-based information changing strategy is conducted to guarantee the algorithm diversity directly in the upper layer through the cross function. Finally, the proposed algorithm is examined in some path-planning environments and compared with other path-planning for verifying its effectiveness and safety.

**Notations:**  $∥\cdot ∥$ represents Euclidean norm. $R^{n}$ is the *n*-dimensional real number space. ⊊ is the non-true subset operator.

## 2. Preliminary Knowledge and Analysis of ABC Algorithm

### 2.1. Basic Conception of Artificial Bee Colony Algorithm

The artificial bee colony algorithm is derived from the honey-harvesting behavior of honey bees. The bee colony is divided into several groups with different tasks and shares the group information to find the optimal solution. The major assignments can be divided into two swarms, namely employed bees and onlookers [21]. The former forages for food sources and searches only in the local region. The number of food sources equals the onlooker bees population. Once the nectar collection is completed, associated bees can become scout bees and repeat the food search within the entire space and bring the new location into the colony. The procedure for mass fundamentals is described below:

A. Employed bee

In charge of the food exploitation by the defined equation as follows:

(1) $$v_{ij}=x_{ij}+\phi \left(x_{ij}-x_{kj}\right)$$

where i≠k, *j* indicates the dimension index from $\left\{1,2,\cdots D\right\}$, and ϕ is a random factor selected from $\left[-1,1\right]$. $X_{i}=\left\{x_{i1},x_{i2},\cdots ,x_{iD}\right\}$ represents the current food source location, *D* is the dimensions number of $X_{i}$, and $v_{i}=\left\{v_{i1},v_{i2},\cdots ,v_{iD}\right\}$ is the location of a new food source searched for by employed bee. Note that if the fitness of the new sources is higher, the memories of employed bees will keep the new position. Otherwise, the previous one is kept to the next iteration until the nectar is substituted.

B. On looker

A bee is looking for suitable food sources by roulette wheel selection, as shown in Equation (2):

(2) $$p_{i}=\frac{fit_{i}}{\sum_{i=1}^{SN}fit_{i}}$$

where $fit_{i}$ is the fitness value of $X_{i}$, and $p_{i}$ the is the probability of selection. Once the target is locked, onlookers are transformed into employed bees and exploit food sources.

C. Scout bee

If a food source cannot be further improved, new nectar would be produced randomly in the searching space, using Equation (3):

(3) $$x_{ij}=x_{\min,j}+random\left(0,1\right)\left(x_{\max,j}-x_{\min,j}\right)$$

where $x_{\max,j}$ and $x_{\min,j}$ are the maximum and minimum search bounds.

### 2.2. The Pros and Cons Analysis of ABC

In ABC, the group evolution strategy can maximize the utilization of convergence resources. For instance, the setting of the nectar harvesting mechanism allows the algorithm to retain a dynamic search ability even in the later period. However, because of this kind of mechanism, a potential optimum may be disturbed, resulting in the algorithm being unable to achieve better local convergence accuracy in the limited number of iterations. To address the question, Li et al. pointed out that the successful experience generated in the evolution can be used to guide the next foraging behavior [24]. The best value is kept throughout the whole process. While Ref. [25] found that the nectar collection is a random process, if different nectars have the same objective value, the search phase of onlookers might be invalid in the evolution. Therefore, they proposed a neighborhood selection method to improve the updated format. In addition, Zhou et al. introduced a multi-elite strategy to increase the guidance in position updating. Moreover, the employed bee and onlooker bee adapt two different search equations [26]. Nevertheless, this approach does not solve the problem of search range overlapping among elites. Regardless of either approach, the fundament is to prevent the loss of optimal solutions with iterations at the cost of the exploration capacity of the algorithm.

The diversity and local convergence accuracy in the later stage is a pair of contradictory factors. A better solution is to explore the search space as much as possible in the initialization and establish an appropriate promotion method in the middle stages of evolution so as to avoid the limitations on diversity caused by the homogeneous potential optimum in the later stages. While the ABC algorithm can deal with the problem by collecting and reusing the converging resources through the division of functional responsibilities into different swarms. These advantages can be utilized, and this article conducts a novel approach incorporated with PSO to address the shortcomings of the ABC algorithm in a dual-community structure. The community can be regarded as a swarm with a specific evolution pattern.

## 3. The Proposed Parasitism-Relation Structure and ParaPA Algorithm

It is worth noting that the improvement on certain parts of the algorithm always comes at the expense of other performance, and this approach can only achieve better application results with specific requirements. However, in most of the modifications, the efficiency of the algorithm does not increase, and the extra parameter setting in the mechanism also asks for higher requirements during the initialization process. While the structure of dual communities can compensate for the defects of each algorithm by establishing the parasitic relationship to integrate the advantages of algorithms. The problem is how to ensure the information interchange and rebuild the evolutionary mechanism based on the relationship to balance the exploration and exploitation ability during the whole process. Hence, the major purpose of the structure is to address the search balance problem.

### 3.1. The Parasitism-Relation Structure

The superiority of the PSO is that it can utilize the best personal and global memories to increase evolution efficiency. This paper establishes the relationship between particle swarms in PSO and bee colonies by the parasitism phenomenon in the biosphere. In previous studies, some researchers have used the symbiosis phenomenon to construct the evolutionary process (symbiotic organisms search algorithm, called the SOS algorithm) [27,28]. Although the same conception of symbiosis is utilized, the understanding of symbiosis is quite different. In essence, the SOS algorithm divides the evolution process into three stages, named mutualism, commensalism, and parasitism, which correspond to the initialization stages for producing diversity, the middle stage for evolving toward the best position, and the last stage for preventing populations from stagnating by generating a random sample, respectively. In terms of modes and the formulation established in SOS, the essence is a DE algorithm with a phased evolution. As the concept of symbiosis described in Ref. [29], to mimic the symbiosis phenomenon, a more appropriate approach is to focus on the swarm relationship rather than individuals. The survival of any swarm is determined by whether it can converge to the final evolution. While in a parasitic relationship, the host has a superior resource, and the inferior party can only attach to the dominant position to keep evolution and competition for a chance to survive. The organism, however, has limited nutrition, which means the exploited position should keep changing throughout the whole process. When the convergence resources are exhausted, the attached particle also loses the right to survive and thus enters a chaotic state. The interpretation of this relation in mathematics is (Note that the multi-path planning is related to the multimodal optimization, which is adapted in this paper.):

**Definition** **1.** Global best. If $\exists x^{*}\in S$, for ∀x∈S where S is the search space with $S\subset R^{n}$, can have $f\left(x\right)\leq f\left(x^{*}\right)$, then the $x^{*}$ is regarded as the global best decision variable in S and $f\left(x^{*}\right)$ is named the global best value.

**Definition** **2.** Multimodal Function. $f^{*}$ is the best value on S based on f, if there are different decision variables $x_{1},x_{2},\cdots ,x_{m}\in S$ where $f\left(x_{i}\right)$(i=1,2,⋯m) are the global best or local best value, the function $f\left(x\right)$ can be named as the multi-peaks function or multimodal function.

Based on Definition 2, the process of finding the best decision variables in *S* based on the multimodal function *f* can be named multimodal optimization.

**Definition** **3.** *Parasitism relationship in multimodal optimization. S is regarded as the living space for the decision variable x. Suppose there are m optima in S, recorded as $\Phi_{1},\Phi_{2},\cdots ,\Phi_{m}$, $\exists S_{1}⊊S$, when the iteration is t and the $S_{1}$ can be recorded as $S_{1}\left(t\right)$, then ∃P, 0<P<1, satisfy*

$$\lim_{t\to \infty }\prod_{i=1}^{m}P\left(\Phi_{i}\in S_{1}\left(t\right)\right)⩾P$$

*P is the convergence probability, which is defined in fuzzy logic. Further, for $\forall S_{2}⊊S$, $S_{1}\neq S_{2}$, for ∀0<δ<1, can satisfy*

$$\lim_{t\to \infty }\prod_{i=1}^{m}P\left(\Phi_{i}\in S_{2}\left(t\right)\right)<\delta$$

*Then called the $S_{1}$ and $S_{2}$ is the parasitism relationship in the living space S.*

**Definition** **4.** *Suppose $\Phi_{i}$ is the i-th optimum in the living space S, if ∃β∈S, ∀δ>0, if $∥\Phi_{i}-\beta ∥<\delta$, then*

$$P\left(\Phi_{i}\in S\right)=1.$$

*If a threshold value is determined, let $0<\theta_{1}<1$, $\exists \theta_{2}$ and $0<\theta_{2}<\theta_{1}$, when ∃β∈S and $∥\Phi_{i}-\beta ∥<\theta_{1}$, then*

$$P\left(\Phi_{i}\in S\right)=\frac{∥\Phi_{i}-\beta ∥}{\left|\theta_{1}-\theta_{2}\right|}.$$

*Further, for ∀β∈S, it always has $∥\Phi_{i}-\beta ∥>\theta_{1}$, then*

$$P\left(\Phi_{i}\in S\right)=0$$

*To summarize, as*

$$P\left(\Phi_{i}\in S\right)=\left\{\begin{array}{c} 1,\;\;\;\;\;\;\;Totally\;convergence\\ 0,\;\;\;\;\;\;\;Totally\;not\;convergence\\ \frac{∥\Phi_{i}-\beta ∥}{\left|\theta_{1}-\theta_{2}\right|},\;Fuzzy\;convergence \end{array}\right.$$

The commensalism and mutualism relationship is described in Appendix A.

**Figure 2:**
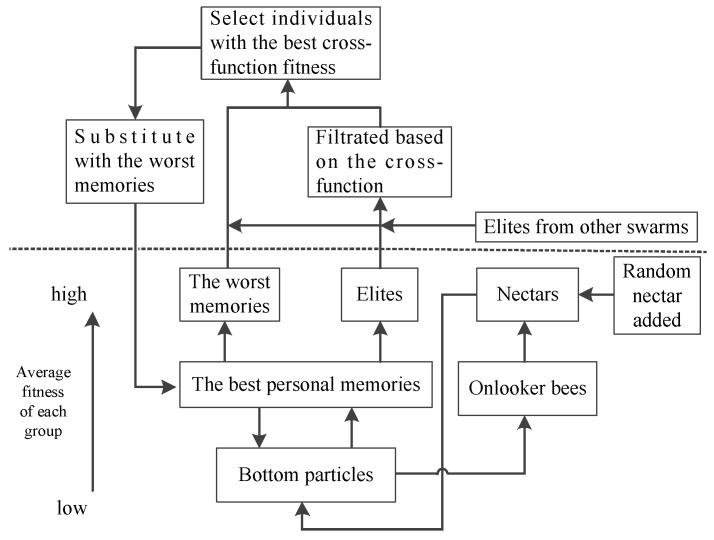
Evolutionary structure of the ParaPA algorithm.

**Figure 3:**
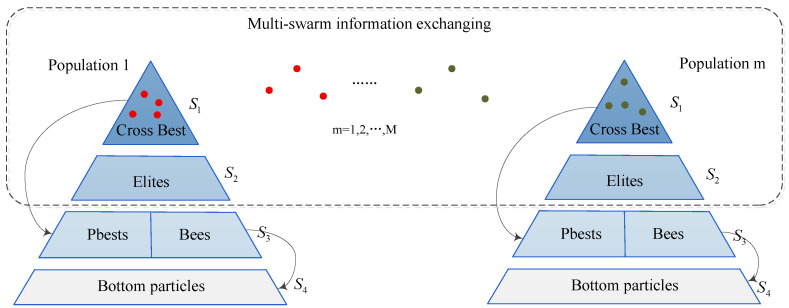
The hierarchical illustration of the ParaPA algorithm with multi-swarm strategy.

In the ParaPA algorithm, the bee colony is regarded as the host part to control the better convergence resources, while the survival of particles in PSO is living in the bottom population, such as the structure, as shown in Figure 2. The life of particles in PSO swarm leeches onto the onlookers and personal best swarms. In other words, the bottom particles cannot find the evolution direction without the instruction from the pbest and bees swarms. If the populations are divided into different hierarchies according to their objective function fitness, the result can be illustrated as in Figure 3. The $S_{4}$ level represents the bottom swarm whose evolution is dependent on $S_{3}$. Meanwhile, the $S_{3}$ level is constructed from $S_{4}$, and they hold exclusive evolution strategies, such as the multi-swarm-elites strategy in pbest swarm and look limitation strategy in the bee colony, which also aims to enhance the algorithm performance in exploitation and diversity, respectively. $S_{1}$ and $S_{2}$ belong to the superior level, which is mainly responsible for exchanging the best information in its population with other populations and also conveying the feedback to $S_{3}$. Hence, the major function of a superior level is to guarantee the information changes among the different populations to avoid evolutionary problems from a monotonous population.

### 3.2. Evolutionary Process of ParaPA

#### 3.2.1. Independent Evolution Stage

A chaotic distribution has been proven to achieve a better statistical property and have faster convergence to the algorithm [30]. To prosper the diversity of the food sources initialization, in this paper, the Logistic map, which is widely used in the chaos-based initialization, is given as follows:

(4) $$x_{n+1}=\mu x_{n}\left(1-x_{n}\right)$$

where *n* is the number of chaotic variables and $x_{0}\notin \left\{0,0.25,0.5,0.75,1\right\}$. The chaotic control parameter $\mu \in \left[3.57,4\right]$. We use μ=4 to produce the chaotic system.

During the initiation, the food resources are selected based on objective fitness, and then all the particles in PSO will transform to onlooker bees to choose the nectar as the global best. Moreover, personal memory is also kept to create more possibilities during the iteration.

#### 3.2.2. The Fusion Stage

Based on the independent evolution, the novel position update incorporated with the bee colony is introduced as follows:

(5) $$V_{id}^{t+1}=\omega *V_{id}^{t}+r_{1}*\left(pbest_{id}^{t}-X_{id}^{t}\right)+r_{2}*\left(onlooker_{id}^{t}-X_{id}^{t}\right)$$

(6) $$X_{id}^{t+1}=X_{id}^{t}+V_{id}^{t+1}$$

where $V_{id}$ is the *d* dimensional velocity of the current particle after *t* iterations, $X_{id}$ is the *d* dimensional location of the current particle after *t* iterations, and the inertial weight *w* is utilized for the exploration ability, changing from $\left[0.1,0.9\right]$ (as shown in Equation (7)) through the evolutionary process, which is a self-regulating method.

(7) $$\omega =\omega_{\max}-\left(\omega_{\max}-\omega_{\min}\right)*\frac{g}{Maxgen}$$

where *g* is the current iterative time, and *Maxgen* is the total number of iterations. $r_{1}$ and $r_{2}$ are two constants, which are taken randomly from 0 to 2 in this paper. The global best is replaced by the best onlooker bee, which is selected by the roulette wheel, as shown in Equation (2).

The limitation of nectar collection is set to three times the number of the population, which means that when all individuals visit the current solution more than three times, the nectar should be abandoned and the onlooker bee on the current nectar would be respawned inside the solution space according to Equation (3). Meanwhile, the original onlooker bees, regarded as the host, compete with the parasitic individuals during the evolution. The host would be directly replaced once a better position appears and the visited number is also restarted. It is worth noting that there is no memory preservation for the onlooker. Originally, the attached individuals would fall into a chaos status when the onlooker is regenerated. However, due to the record of the personal best position, the parasitic individual can still move toward the best position in its memory after losing the global guidance so that the algorithm can keep the exploitation capacity in the middle stage of evolution and avoid the waste of iterations.

#### 3.2.3. Interaction Stage with the Multi-Swarm Elite Strategy

In order to enhance the diversity of the algorithm, this paper adopts a multiple swarm parallel strategy. Each swarm is an independent biosphere, and its parasitic relationships are bound in the biosphere, which is not influenced by other environments. To avoid overlapping regions of the host during the convergence, each population takes out the best memory to join the mixing pool after each evolution (as illustrated in Figure 2). The quality of all elites in the mixing pool is judged by the cross function instead of the previous objective function. Furthermore, the worst individuals are usually discarded via evolution, but the amount of information carried by them can create substantial value in multimodal situations. For the consideration of diversity, the worst individuals in the personal best memory are also mixed with the individuals selected from the mixed pool who have better cross function values and then re-screened by the related objective function. Finally, the individuals with the worst fitness in each swarm would be replaced by those chosen memories in the next iteration (the pseudocode of multi-swarm elites selection is shown in Algorithm 1).
**Algorithm 1** Multi-swarm-elite selection strategy.**Input**: Select elites from the best personal memories in each sub-swarm based on the objective functions and add them to the mixed pool.**Steps**: **for** each sub-swarm              1. Select several worst personal memories in each sub-swarm (suppose the number is *w*).              2. Choose the best individuals from the mixed pool according to the cross fitness. Then, mix the worst *w* individuals into with them and remove other elites in the pool.              3. **for** each individual in the mixed pool                         Evaluated it by the cross function                              **if** current individual is better                                      Record it in the pool-output group (In this paper, the population of pool-output group is equal to 1)                              **end**                   **end**              4. Replace the worst individuals in sub-swarm by the pool-output group into next iterations             **end****Output**: Each sub-swarm with elites who has the best cross fitness. To note that the replaced individuals always keep the best memories.

## 4. Problem Formulation and the Strategies for Path Planning

In this paper, several static obstacles are listed in the environment along with some premises and assumptions, shown as follows:

(1) Global path planning is the main target in this paper, which means all obstacles are known before algorithm execution.
(2) The environment of path planning is built in a 2D workspace. The path planning will consider obstacle avoidance without height.
(3) If the planning path can avoid the obstacles successfully under environmental constraints, it means the algorithm has the ability to build a safe path in the static condition. Hence, the physical characteristics of the robot are not considered in this paper.
(4) In the static condition, the robot speed is constant.

### 4.1. Workspace Formulation

**Figure 4:**
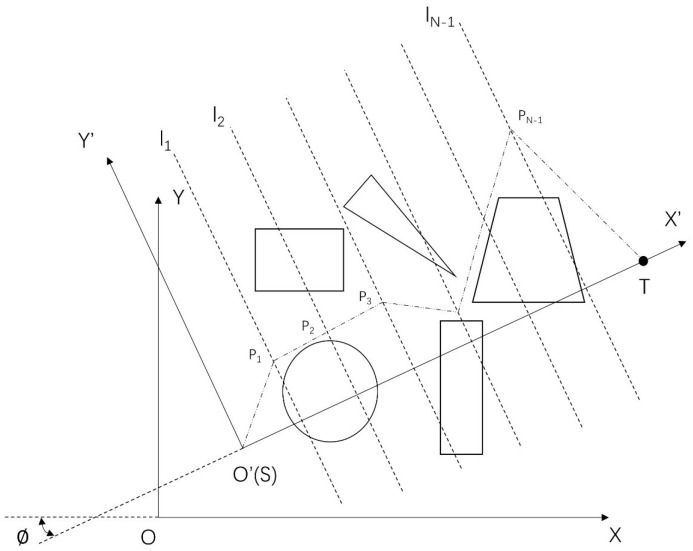
The coordinate transformation for path planning.

Normally, the path planning question has two or three decision variables. Although it can enrich the diversity in the evolution process, the convergence efficiency of the algorithm will be significantly reduced in the face of complex obstacle situations. Therefore, a new coordinate is applied to connect the start and target positions, as shown in Figure 4. Then the new $x^{\prime }$-axis is divided into *N* segments averagely, which means the *x* dimension is fixed in the new coordinate. While the *y* dimension is optimized along the parallel line $L_{i}$ (i=1,2,…,N−1), which is vertical to the new $x^{\prime }$-axis. Hence, the planning path can be presented as ($S_{1},S_{2},S_{3},\ldots ,S_{n}$), where n=1,2,…,N and the start and target point are fixed. The corresponding transformation formula is shown in Equation (8).

(8) $$\left[\begin{array}{c} x^{\prime }\\ y^{\prime } \end{array}\right]=\left[\begin{array}{cc} \cos\phi & -\sin\phi \\ \sin\phi & \cos\phi \end{array}\right]\left[\begin{array}{c} x\\ y \end{array}\right]+\left[\begin{array}{c} Start_{x}\\ Start_{y} \end{array}\right]$$

where the $\left(x,y\right)$ and $\left(Start_{x},Start_{y}\right)$ belong to the original cartesian coordinate frame. ϕ is the rotation angle to the new frame. Moreover, the map boundary is changed. For instance, in Figure 4, the new $y^{\prime }$-axis intersects with the original *y*-axis. For any point on the parallel line $L_{i}$, the boundary of $y^{\prime }$ in the new frame is determined as follows:

(9) $$y{}_{\max}^{\prime }=\left\{\begin{array}{c} \frac{x_{i}}{\sin\phi },x_{i}<x_{m}\;and\;y_{i}<y_{m}\\ \frac{ul_{y}-y_{i}}{\cos\phi },x_{i}>x_{m}\;and\;y_{i}>y_{m} \end{array}\right.$$

(10) $$y{}_{\min}^{\prime }=\left\{\begin{array}{c} -\frac{y_{i}}{\cos\phi },x_{i}<x_{m}\;and\;y_{i}<y_{m}\\ -\frac{ul_{x}-x_{i}}{\sin\phi },x_{i}>x_{m}\;and\;y_{i}>y_{m} \end{array}\right.$$

where $ul_{y}$ and $ul_{x}$ are the upper limits of the *y*-axis and *x*-axis, respectively. Further, the segment points are located on the $x^{\prime }$-axis, such as $P_{i}\left(x_{i},y_{i}\right)$, with respect to the original frame. For any points that go over the boundary during the evolution, the whole path $\left(S_{1},S_{2},S_{3},\ldots ,S_{n}\right)$ should be regenerated in the solution space. To satisfy the safety issue, each segment $S_{n}$ should not intersect with any obstacles, and all the objective functions are calculated on the original cartesian coordinate frame.

### 4.2. Design of Objective Function

The quality of a path is examined by the fitness value. In the previous study, only feasible paths were evaluated, and information on infeasible paths was neglected [31], which makes it difficult to take the valid yet easily ignored information into account for global planning. Therefore, in this paper, different objective functions are used for the assessment of feasible and infeasible paths, respectively. According to the previous definition, when any segments are overlapped with obstacles, the current path is regarded as an infeasible solution. For the evaluation of infeasible paths, the penalty for worse individuals should be increased so that the evolution of the infeasible solutions can move toward the local best, which has the least intersecting segments. While for the feasible path, when the speed is constant, the total path length should be considered primarily. Since the idealized robot model is adopted in this paper, the kinetics effects are not considered, but the path should be smoothed as much as possible during planning. Based on the aforementioned hypothesis, a path is composed of *N* segments and N+1 nodes; then the evolutionary variable can be represented as ${\mathrm{path}}_{i}$$\left(p_{1},p_{2},\cdots ,p_{N+1}\right)$ where $p_{1}$ should be the fixed start point, and $p_{N+1}$ is the target point. Hence, the length of the path can be calculated as

(11) $$\begin{array}{cc} f_{1} & =\sum_{n=1}^{N}\sqrt{{\left(x_{n+1}-x_{n}\right)}^{2}+{\left(y_{n+1}-y_{n}\right)}^{2}}\\ \; & =\sum_{n=1}^{N}\sqrt{{\left(\frac{d\left(x{}_{1}^{\prime },x{}_{N+1}^{\prime }\right)}{N}\right)}^{2}+{\left(y{}_{n+1}^{\prime }-y{}_{n}^{\prime }\right)}^{2}} \end{array}$$

where $x_{n}$ and $y_{n}$ are the original coordinates of node $p_{n}\left(x_{n},y_{n}\right)$. $d(x_{1}^{\prime },x_{N+1}^{\prime })$ is the distance from the start point to the target along the $x^{\prime }$-axis and $y_{n}^{\prime }$ is the transformed coordinates.

The smooth function can be defined as follows:

(12) $$\begin{array}{c} f_{2}=\pi -\sum_{j=2}^{N}\cos^{-1}\left[\frac{\left(x_{j}-x_{j-1}\right)\left(x_{j+1}-x_{j}\right)+\left(y_{j}-y_{j-1}\right)\left(y_{j+1}-y_{j}\right)}{\sqrt{{\left(x_{j}-x_{j-1}\right)}^{2}+{\left(y_{j}-y_{j-1}\right)}^{2}}\times \sqrt{{\left(x_{j+1}-x_{j}\right)}^{2}+{\left(y_{j+1}-y_{j}\right)}^{2}}}\right] \end{array}$$

which represents the summation of the angles between every connected segment. It starts from the second point and calculates the angle between the first and second segments, et cetera. The small $f_{2}$ value means a small direction change in each turn, representing a better path.

The overall objective function for the feasible function is expressed as follows:

(13) $$F_{feasible}=\frac{1}{f_{1}}+kf_{2}+C$$

where *k* is the weight of smoothing and a higher *k* can obtain a smoother path, but the diversity might be affected. *C* is the feasible and practical reward parameter which is a positive number that is not greater than the maximum path length in the current environment.

For the infeasible condition, except for the length of the path, the ratio of infeasible segments and the ratio of the infeasible path over the total length are involved in the estimation as follows:

(14) $$f_{3}=\frac{N_{\inf}}{N}$$

(15) $$f_{4}=\sum_{i=1}^{N_{\inf}}\frac{d_{obs}^{i}}{f_{1}}$$

where the $N_{inf}$ is the number of infeasible segments and $d_{obs}^{i}$ is the overlapping distance between each infeasible segment and the obstacles. The objective function for the infeasible function is expressed as follows:

(16) $$F_{infeasible}=\frac{1}{wf_{1}}+\frac{1}{f_{3}+f_{4}}$$

where *w* is the weight to adjust the influence of total infeasible path length.

### 4.3. Design of the Cross Function

The main purpose of the multimodal strategy is to increase the efficiency of path planning, which can provide several collision-free paths from the start position to the target. Once a path falls into an emergency condition, the potential solution can change to another option immediately. The target and initial point for the robot are already known before the real application. The motion of the robot is from its current position to reach the next subsequent segment, and the process will continue until it reaches its goal position. Hence, segments of the robot should not intersect with obstacles or any other potential solution. For the design of the cross function, the objective is to generate a constraint that minimizes the arrival time for each planned path, and meanwhile, each segment cannot overlap with other paths as much as possible. Based on the above analysis of the algorithm, the best individuals in each sub-swarm are first mixed, and the cross fitness of each population elite is calculated based on the following equation, Equation (17), assuming that the number of elites is *E*.

(17) $$cross\;function=\sum_{e=1}^{E}\sum_{n=1}^{N}Cp_{n}^{e}=\sum_{e=1}^{E}\sum_{n=1}^{N}\left(\begin{array}{ccc} Cp_{1}^{1} & \cdots & Cp_{N}^{1}\\ \vdots & \ddots & \vdots \\ Cp_{1}^{E} & \cdots & Cp_{N}^{E} \end{array}\right)$$

where the index *n* is the *n*-th segment in the current evaluated path, the index *e* is the *e*-th elite in the other sub-swarms, and $Cp_{n}^{e}$ is the cross value, which represents the cross fitness of individual *i* and interactive elite *e*. The cross function should be applied to each sub-swarm, and the cross evaluation should go through the sub-swarms except for the current swarm. Moreover, only the elite with the lowest number of segment intersections will be selected and mixed again with the worst individuals in each group memory. After that, all individuals are examined by the cross function again, and the elite who has the greatest difference from others in the mixed group is selected in the next iteration. It is worth noting that all mixing processes are evaluated only by cross fitness to avoid the influence of other factors.

### 4.4. Multimodal Path Planning Strategy

Multimodal path planning is designed to find multiple paths in a single run. When an emergency occurs on a path, the potential solution can be immediately changed to another option, thus addressing the inefficiencies of traditional path planning. Different from the scheme of a bug algorithm robot walking around obstacles when encountering obstacles and walking along a straight line without encountering obstacles [32], the multimodal path planning strategy adopts the segmented method as described in Section 4.1. Each node position of the segmented ($P_{1},P_{2},...,P_{N}$) corresponds to a dimension of the variable; then the paraPA algorithm is used to find multiple paths. The optimal value with the best fitness that each swarm finally converges to is a global optimal path, while coevolution between multiple swarms ensures the diversity of the paths.

During the demonstration, we found that normally, there are one or two segments that are infeasible, causing the failure of the whole path, which is especially common among the superior populations at the late stage of convergence. To address this problem, this paper proposed a multi-path-based reverse planning strategy, which can be regarded as a complementary strategy for the replacement strategy to nectar sources in the bee colony. If the replaced nectar source only has one or two infeasible segments, the reverse planning strategy is triggered. Specific details of this strategy is shown in Algorithm 2:
**Algorithm 2** Multi-path-based reverse planning strategy.**Input**: Paths with segments that are infeasible.**Steps**: **for** each infeasible path              1. Find the index of the non-viable section (suppose $S_{i}$ is the infeasible segment).              2. Go through the pbest group to check.                      **if** there is a feasible solution in the same section ($S_{j}$)                             Examine the segments from $S_{j}$ back to the start segment.                                  **if** segments from start to $S_{j}$ in $pbest_{p}$ are all feasible                                           Record the corresponding node in this $pbest_{p}$ and replace the corresponding positions in nectar.                                   **end**                        **else**                              Trigger the random generation process of nectar.                        **end**                  **end****Output**: New feasible paths.

## 5. Experiments and Analysis

### 5.1. Environments and Comparison of Algorithms

In recent years, researchers have been using the population intelligence approach for path planning [33]. In order to examine the effectiveness of the ParaPA algorithm on different obstacle situations, three different types of scenarios are conducted in this paper to examine the robustness, efficiency, and convergence performance of the algorithm. The first is 20 × 20 maps, which are used to detect the sensitivity of algorithms to different types of obstacles. Second, rectangles are randomly generated in 50 × 50 maps with various sizes as obstacles, which is applied to test the adapted capacity of the algorithm under the complex environment. The third category, an actual scenario simulation, named Small Cultural Complex, is adopted as the application environment built-in 100 × 100 maps. The Small Cultural Complex is a new cultural industry model, which achieves different cultural function requirements by transforming the inside construction of the building, and during the transformation, mobile robots would take charge of the functional equipment transport, such as stage props or lighting. In this paper, the exhibition, sports activity, and theatrics functions are modeled, respectively. Meanwhile, to further show the advantages of the multi-swarm strategy, this paper uses PSO, CPSO, WPSO, ABC, and ACO algorithms for comparison experiments. Furthermore, MSPO and MABC are applied to test the effect of the proposed structure, also to evaluate the algorithm in terms of planning effectiveness, stability, etc.

### 5.2. Experimental Settings

For all algorithms, the data are calculated from 1000 runs, and the maximum evolution times are 200. For parameters setting in the PSO algorithm, the population size for each swarm is NP=50, and the maximum and minimum evolution steps are Vmax=2 and Vmin=−2, respectively. The acceleration factors are accepted as a constant 1.49445 in this paper. Specifically, for the WPSO, the inertia weight *w* is linearly changed from 0.1 to 0.9. Otherwise, the *w* is adapted as a constant 0.7. While for CPSO, the chaos-based initialization is utilized with a logistic chaos map to enrich the algorithm diversity where μ=4 is adapted. For the parameter setting of the ABC algorithm, the look limitation is adapted as 5×NP, which means all individuals in the swarm should visit the nectar more than five times, then the nectar can be replaced. In ABC and MABC, the size of onlookers is equal to the size of employed bees, but in the ParaPA algorithm, this number is equal to NPNP55. Moreover, the number of elites in each swarm is taken as 10 while 3 of them will be selected in the cross-verification process. Moreover, the number of elites in each swarm is taken as 10, while 3 of them will be selected in the cross-verification process. The start points for 20 × 20 and 50 × 50 maps are set at point $S\left(0.5,0.5\right)$ and the target points are $E_{20}\left(19.5,19.5\right)$ and $E_{50}\left(49.5,49.5\right)$, respectively. While for 100 × 100 maps, the start points are set to $S_{100}\left(3.5,3.5\right)$, and the target points are $E_{100}\left(99.5,99.5\right)$.

### 5.3. Results Representation and Analysis

#### 5.3.1. Scenario 1: Path Planning on 20 × 20 Maps with Different Types of Obstacles

**Figure 5:**
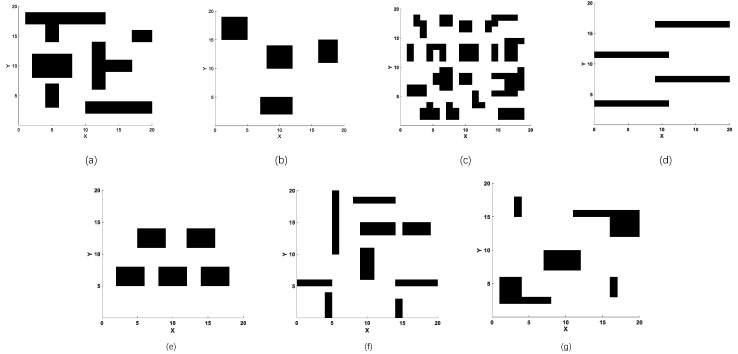
Set of test 20 × 20 maps for the first scenario in which (**a**–**g**) show the different distributions of obstacles on 20 × 20 maps.

In the first case, different obstacles are constructed in a 20 × 20 map, as shown in Figure 5a–g to examine the sensitivity of the algorithm on obstacle shapes. The length of each chromosome is set to 12 for all algorithms. Results are shown in Table 1, and all the best values are highlighted in bold. First, by comparing with the particle swarm algorithm in the original coordinate, it can be clearly verified that the performance of the algorithm with the new coordinate system is significantly improved, but the problem is also ParaPA. As can be seen in the test of Map 4, the mean and variance under the original coordinates are the best, which means that the new coordinate system is inferior to dealing with the S-shaped trajectory planning. Because one dimension is fixed in the new coordinate system, the changeable range for each individual is less than the original coordinate. Hence, when in an environment with several continuous transverse obstacles, such as Map 4, the path generated by the algorithm is relatively monotonous, resulting in a worse convergence effect. It is worth noting that the best value of optima, as well as the worst individual in Table 1, are not in the original coordinate system, indicating that the algorithm in the new coordinate system could be superior during the evolution once the algorithm can find a feasible sample as soon as possible. From the results of other maps, the ParaPA algorithm has the best performance in 1000 runs, and the final convergence achieved the shortest path length in most scenarios. While the algorithm with the best stability is ACO, its evolutionary process is easily stagnant, resulting in unsatisfactory final convergence results. In comparison, the multi-swarm strategies, such as MABC and ParaPA algorithm, can increase the path diversity in a complex environment, and the proposed structure can be better exploited locally for better convergence results.

Table 2 shows the success rate. Note that the multiple population strategy proposed in this paper, combined with the parasitic relationship of two communities, can increase the probability of a successful search for the special continuous transverse obstacles in Map 4. In most cases, the algorithm can successfully find a suitable path. The results, however, are different in terms of the algorithm’s efficiencies combined with data from Table 1. The structure of ParaPA can utilize the population differences in multiple swarms and transform them into the diversity of path samples so as to improve the effectiveness of the algorithm, and the diversity of the algorithm is expressed by the individual disparity in the population in the following:

(18) $$DI^{G}=\frac{1}{NP}\sqrt{\sum_{i=1}^{NP}{∥X_{i}^{G}-\frac{1}{NP}\sum_{j=1}^{NP}X_{j}^{G}∥}^{2}}$$

where DI is the individual diversity [34], *G* means generation, and NP is the number of populations.

#### 5.3.2. Scenario 2: Path Planning on 50 × 50 Maps with Randomly Generated Rectangles as Obstacles

**Figure 6:**
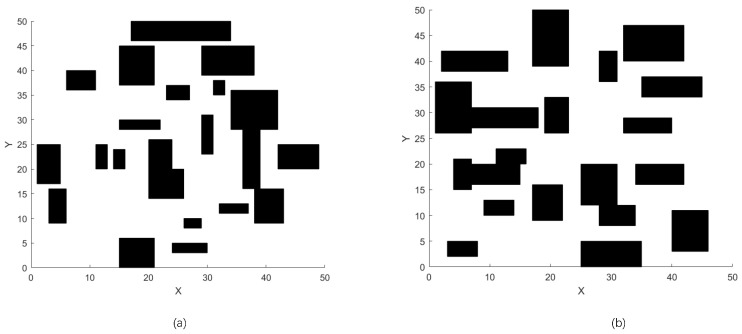
Set of test 50 × 50 maps for the second scenario in which (**a**,**b**) show the different distributions of obstacles on 50 × 50 maps.

While the merits of diversity are not obvious in the simple condition. To verify the convergence and searching capacity of different algorithms, more complex environments are built with randomly generated rectangles in 50 × 50 maps, as shown in Figure 6. In terms of the success rate from Table 2, ParaPA, ABC, and MABC are the only algorithms that can guarantee the finding of a feasible path, while Table 3 shows the convergence performance of the algorithm in the 50 × 50 environments. It is worth noting that the most stable algorithm is MABC, while the ParaPA algorithm makes some stability sacrifices in order to obtain a better search breadth in return for more accurate local convergence. This can be seen from the best value of optima in each map, indicating that the ParaPA algorithm has a stronger local searching ability. The standard deviation reflects the stability of the algorithm’s performance in a scenario, while the value of DI is an indicator of the diversity of the algorithm. In Table 4, the above algorithms with better performance are selected to compare the diversity. The standard deviations of CPSO and WPSO are larger compared with others. Meanwhile, from the performance of DI, the algorithm does not construct more feasible paths during chaos search, resulting in poorer sample diversity. The proposed structure performs chaos at the bottom to create more feasible paths, and the superior individuals are responsible for fine-tuning the optima. Combining the optima value in Table 3 and the DI performance in Table 4 shows the capacity of the ParaPA algorithm both in local convergence accuracy and global exploration.

**Figure 7:**
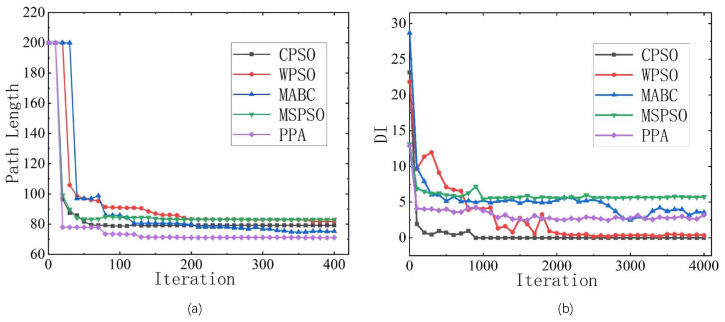
Convergence comparison on 50 × 50 maps where (**a**) is the path length change curve during iteration and (**b**) shows the curves of the diversity of individuals (DI) within 4000 iterations.

The convergence curves in 50 × 50 maps for the compared algorithms are shown in Figure 7 to observe the convergence variation during an evolution where the maximum number of evolutions is set to 4000. The frequency and magnitude of the changes in the path length are small enough to be ignored after approximately 300 iterations. Hence only the first 400 iterations are taken into account, as the results shown in Figure 7a. It can be seen that all algorithms complete the planning for a feasible path within 60 iterations, after which the best value is planned locally around it, and the changing magnitude is reduced. Note that the best path is recorded based on the parasitism swarm, indicating that the best position has been locked by the inferior population at around 400 iterations. After that, what needs to be performed for the algorithm is to maintain the diversity to create more possibilities for path convergence. The DI is measured in 4000 generations to observe the tendency, as shown in Figure 7a. It can be seen that it is difficult to maintain a stable diversity for CPSO as well as WPSO, which means that their convergence tendency will fall into a unified position. ParaPA and MABC are more stable in terms of diversity performance.

#### 5.3.3. Scenario 3: Path Planning for Real Application on 100 × 100 Maps

**Figure 8:**
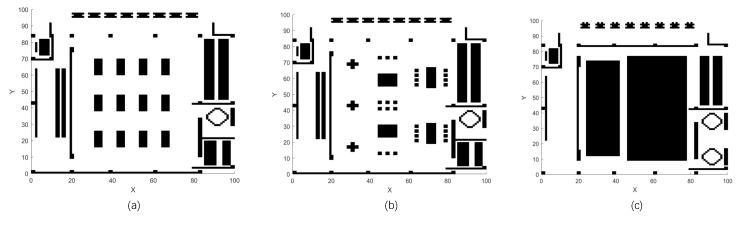
Real application on 100 × 100 maps of the Small Cultural Complex where (**a**–**c**) are the layouts corresponding to the function of the exhibition, sports, and performance, respectively.

In practical map applications, as illustrated in Figure 8 [35], this paper chooses algorithms with a better performance in the above test to make a comparison.

**Figure 9:**
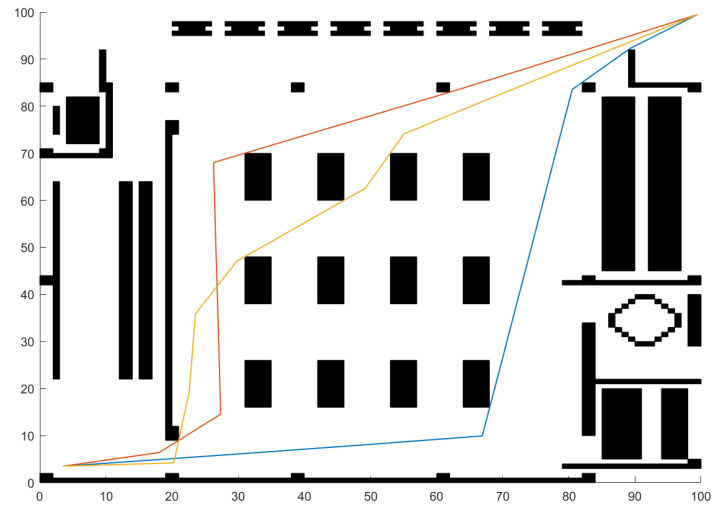
Path diagram in which colorful lines represent three routes obtained by a signle run on a 100 × 100 map.

The results in Table 5 and Table 6 show that the ABC series algorithm is able to create more sample diversity but hardly converts the recourses to the final convergence accuracy. For the ParaPA algorithm with a parasitic relationship, the bottom particles can guarantee the search ability, but the “nutrients” they can pass to the superior layer would be reduced. It can be solved by building multiple population interactions to increase the diversity in the upper layer population directly. From the test on Maps 2 and 3, MABC has a better performance in terms of stability. In other words, MABC does not have any internal mechanism to refine the local search, so the higher stability can be regarded as the outcome at the sacrifice of the exploitation ability as the optima result in Table 5. Consequently, the proposed algorithm has a better performance on most of the measurements, but how to choose an algorithm should consider the specific requirements of the real application. Furthermore, in order to show the results of multimodal path planning more intuitively, we take map (a) in Figure 8 as an example and show the multiple paths planned by the single-run algorithm in Figure 9. In a single run, it generates the three paths with the best fitness values, as shown in Figure 9.

## 6. Conclusions

Primarily, this paper introduces a dual-community-based evolutionary algorithm model that mimicked parasitic relationships in the biosphere. In the established structure, the bottom particles guarantee the algorithm diversity, while the superior community controls the better convergence resources and is responsible for completing the local convergence. Meanwhile, a multiple population strategy is conducted that is utilized to build information interaction channels among superior populations and directly shares the best information contained in superior populations. Finally, based on the above algorithm structure, ParaPA is proposed to solve the path planning problem. Meanwhile, we design a cross function to filter the high-level interactive information through the proposed multiple swarm framework so as to ensure path diversity in the superior population. From the comparison of the algorithm diversity and the average length of paths, the proposed approach is able to produce more path possibilities by using the structure, achieving a better solution in regard to path length.

Compared with traditional path planning strategies, such as A* algorithm, because of the characteristics of ABC and PSO algorithms, the ParaABC algorithm can effectively solve high-dimensional path planning problems. In the process of algorithm operation, once the target point is unreachable, the proposed algorithm can discard the current path directly or re-plan the infeasible section according to prior knowledge, thus saving performance loss. In addition, the ParaABC algorithm can plan multiple optimal paths through a single operation. Compared with the popular method of neural networks in recent years, the proposed method only needs to adjust a few parameters and has low equipment requirements. However, although the performance of ParaABC is improved compared with similar algorithms, it still faces the problems of diversity disappearance and premature convergence.

In the future, the three symbiosis-relation-based evolutionary structures introduced in this paper could be studied for different applications. Under the mutualism relationship, the convergence probabilities of multiple populations are equivalent, similar to the ring topology, which is suitable for multimodal or multi-objective optimization problems. For the commensal relationship, the interspecies relationship is inclined to a competition that has a continuous dynamic optimization capability. While in the parasitic relationship, where the convergence will tend to be homogeneous during the evolution, the avoidance of the premature problem is required. Note that the symbiosis relationship construction establishes an information bridge among multiple populations rather than a specific individual. Meanwhile, the development of symbiosis-relation-based evolutionary conception for different applications through the adapted framework is an interesting field that may be undertaken in the future.

## Figures and Tables

**Table 1:** Convergence performance comparison for scenario 1 on 20 × 20 maps.

MAPs		ParaPA	PSO(ori)	PSO	CPSO	WPSO	MPSO	ACO	ABC	MABC
Map1	Mean	**28.531** ^1^	32.311	30.059	29.919	30.079	32.644	38.002	28.667	28.583
	Std	0.143	4.245	2.767	1.851	1.719	8.640	0.063	0.170	**0.042**
	Worst	29.825	58.069	89.130	40.225	38.921	212.317	40.000	31.328	**28.802**
	Optima	**28.455**	29.228	28.459	28.459	28.455	28.475	38.000	28.480	28.480
Map2	Mean	**27.024**	32.526	28.041	28.007	28.009	27.496	38.032	27.044	27.033
	Std	**0.005**	5.596	1.116	1.077	1.015	0.492	0.251	0.018	0.006
	Worst	27.098	53.125	36.749	34.031	32.926	30.317	41.000	27.173	**27.060**
	Optima	**27.022**	27.295	27.022	27.022	27.022	27.025	37.000	27.022	27.022
Map3	Mean	**28.265**	39.905	31.822	30.668	30.541	30.171	38.000	29.777	28.670
	Std	0.419	7.300	6.352	2.084	1.885	2.056	**0.000**	1.542	0.571
	Worst	**30.495**	84.383	99.917	41.312	39.035	42.676	38.000	37.597	33.039
	Optima	**27.848**	29.482	27.882	27.900	27.914	27.994	38.000	28.102	28.170
Map4	Mean	57.444	**47.617**	72.308	52.553	NaN	82.221	81.612	84.206	73.934
	Std	14.674	**3.723**	14.388	5.138	NaN	16.069	17.741	15.514	13.658
	Worst	96.037	69.024	108.846	**60.659**	NaN	114.122	150.000	113.900	114.461
	Optima	41.738	40.665	46.061	**38.334**	NaN	55.814	50.000	56.989	48.293
Map5	Mean	**28.922**	36.411	31.515	31.430	31.538	29.455	38.004	30.795	29.431
	Std	1.795	7.086	2.341	2.370	2.238	1.845	**0.089**	2.635	1.627
	Worst	33.343	69.188	41.791	42.173	39.855	36.349	40.000	39.659	**32.952**
	Optima	27.290	27.828	27.297	27.302	27.295	**27.275**	38.000	27.376	27.395
Map6	Mean	**28.482**	37.455	29.827	29.868	29.787	29.600	38.000	29.899	29.117
	Std	0.379	6.885	1.434	1.276	1.017	1.598	**0.000**	0.532	0.119
	Worst	**29.487**	63.969	53.169	37.791	37.399	73.792	38.000	32.878	29.724
	Optima	**27.982**	29.696	28.046	28.001	28.041	28.421	38.000	28.936	29.111
Map7	Mean	30.918	37.452	33.140	33.169	32.956	32.700	38.038	33.414	**30.018**
	Std	1.916	3.087	3.128	3.238	3.036	2.730	**0.273**	2.795	0.388
	Worst	37.353	54.301	47.069	46.973	42.344	**29.084**	40.000	40.827	34.502
	Optima	29.139	29.594	29.151	**29.127**	29.136	42.175	38.000	29.385	29.285

^1^ The bold represents the best value.

**Table 2:** Success rate on various maps.

	Maps	ParaPA	PSO(ori)	PSO	CPSO	WPSO	MPSO	ACO	ABC	MABC
Maps 20 × 20	Map1	1	0.988	0.998	0.998	0.999	0.998	1	1	1
	Map2	1	1	1	1	1	1	1	1	1
	Map3	1	0.493	0.871	0.94	0.944	0.966	1	1	1
	Map4	0.499	1	0.07	0.041	0	0.04	0.83	0.016	0.237
	Map5	1	1	1	1	1	1	1	1	1
	Map6	1	0.361	0.944	0.928	0.926	0.995	1	1	1
	Map7	1	1	0.966	0.969	0.936	1	1	1	1
Maps 50 × 50	Map1	1	0.131	0.616	0.794	0.617	0.98	1	1	1
	Map2	1	0.352	0.958	0.793	0.947	0.997	0.836	1	1

**Table 3:** Convergence performance on 50 × 50 maps.

MAPs		ParaPA	PSO(ori)	PSO	CPSO	WPSO	MPSO	ACO	ABC	MABC
Map1	Mean	**74.776**	94.889	87.125	96.964	86.833	80.842	160.233	82.594	79.882
	Std	3.005	21.593	24.458	39.288	20.584	7.851	22.051	3.939	**1.778**
	Worst	103.862	200.684	295.924	315.496	219.431	223.645	266.000	106.806	**86.220**
	Optima	**70.107**	72.954	72.299	73.702	72.403	70.300	114.000	74.303	74.052
Map2	Mean	**76.558**	91.572	83.963	97.128	85.062	78.558	167.916	80.015	78.372
	Std	2.671	14.287	13.566	38.198	16.207	4.347	25.126	2.818	**0.981**
	Worst	**83.936**	152.965	255.782	299.138	238.817	105.914	320.000	96.480	87.981
	Optima	71.287	71.959	72.946	74.968	73.867	**71.361**	116.000	75.309	75.876

**Table 4:** Diversity of individuals (DI) on 50 × 50 maps.

50 × 50 Maps	ParaPA	CPSO	WPSO	MPSO	MABC
Map1	**8.897**	5.073	4.279	6.776	7.021
Map2	**9.338**	7.523	5.618	7.013	5.077

**Table 5:** Convergence performance on 100 × 100 maps.

100 × 100 Maps		ParaPA	CPSO	MPSO	ABC	MABC
Map1	Mean	**147.727**	157.103	155.820	167.317	161.270
	Std	5.702	8.130	7.914	7.717	**4.940**
	Worst	**168.755**	203.790	198.764	206.522	177.713
	Optima	**141.006**	142.508	142.262	148.932	148.287
Map2	Mean	**145.878**	154.001	153.771	161.662	157.390
	Std	9.356	10.786	10.398	5.013	**4.021**
	Worst	347.053	263.723	347.306	188.500	**169.377**
	Optima	**139.750**	141.253	141.306	145.790	145.765
Map3	Mean	**166.445**	171.323	176.595	171.036	169.858
	Std	18.047	2.486	12.6525	4.1758	**0.481**
	Worst	554.350	175.755	335.206	255.509	**170.056**
	Optima	**165.459**	168.992	165.795	168.958	169.838

**Table 6:** Diversity of individuals (DI) on 100 × 100 maps.

100 × 100 Maps	ParaPA	CPSO	MPSO	ABC	MABC
Map1	7.171	6.973	9.249	12.728	**13.409**
Map2	5.601	6.034	8.104	13.430	**14.664**
Map3	4.078	8.424	9.129	**17.583**	10.043

## Data Availability

Not applicable.

## Appendix A

According to the description in [29], the commensalism and mutualism relationship should be defined as follows. All the relationships are defined under the multimodal optimization condition.

Commensalism relationship: The weaker population should give resources to the superior population while it is not affected by the interaction.

Mutualism relationship: The evolutional process will not be interrupted by others among two or more populations and can obtain benefits from group interaction. In other words, each evolution is independent while the information changing among different populations can contribute to their convergence.

We define the relationships in mathematics as:

**Definition** **A1.**
*Suppose there are m optima in S, recorded as $\Phi_{1},\Phi_{2},\cdots ,\Phi_{m}$. There exists $S_{1}⊊S$, $S_{2}⊊S$, $S_{1}\neq S_{2}$. In the t-th iteration, $S_{1}$ and $S_{2}$ are recorded as $S_{1}\left(t\right)$ and $S_{2}\left(t\right)$. If the following function is true*

$$\lim_{t\to \infty }\prod_{i=1}^{m}P\left(\Phi_{i}\in S_{1}\left(t\right)\right)>\lim_{t\to \infty }\prod_{i=1}^{m}P\left(\Phi_{i}\in S_{2}\left(t\right)\right)$$

*then the $S_{1}$ and $S_{2}$ is the commensalism relationship in the living space S.*

**Definition** **A2.**
*Suppose there are m optima in S, recorded as $\Phi_{1},\Phi_{2},\cdots ,\Phi_{m}$. $\exists S_{1}⊊S$, when the iteration is t and $S_{1}$ can be recorded as $S_{1}\left(t\right)$, then $\exists P_{1}\left(t\right)$, $0<P_{1}\left(t\right)<1$, satisfies*

$$\lim_{t\to \infty }\prod_{i=1}^{m}P\left(\Phi_{i}\in S_{1}\left(t\right)\right)=P_{1}\left(t\right)$$

*and for $\exists S_{2}⊊S$, $S_{1}\neq S_{2}$, $\exists P_{2}\left(t\right)$, $0<p_{2}\left(t\right)<1$ satisfies*

$$\lim_{t\to \infty }\prod_{i=1}^{m}P\left(\Phi_{i}\in S_{2}\left(t\right)\right)=P_{2}\left(t\right)$$

*For ∀δ>0, always have*

$$\lim_{t\to \infty }∥P_{1}\left(t\right)-P_{2}\left(t\right)∥<\delta$$

*called the $S_{1}$ and $S_{2}$ is the mutualism relationship in the living space S.*
